# Supplementary material for: Incidence Trend of Follicular Lymphoma in Taiwan Compared to Japan and Korea, 2001–2019
Source: J Clin Med. 2023 Feb 10;12(4):1417. doi: 10.3390/jcm12041417 (PMC9963002; doi:10.3390/jcm12041417)
Supplement: Supplementary file 1 [file jcm-12-01417-s001.zip › jcm-2178357-supplementary.pdf]

Supporting Information

Table S1. Data source

Database	Year	Area
TCR Database	2002-2007	Taiwan. Available from: https://onlinelibrary.wiley.com/doi/full/10.1002/cam4.1762#cam41751-tbl-0002 (published Oct 2018)
TCR annual report	2008-2019	Taiwan. Available from: https://www.hpa.gov.tw/Pages/List.aspx?nodeid=269 (released Feb 2022)
Resident Population, Taiwan	2008-2019	Taiwan. Available from: https://www.moi.gov.tw/files/site_stuff/321/1/month/m1-06.ods
NCR	2001-2008	Japan. Available from https://onlinelibrary.wiley.com/action/downloadSupplement?doi=10.1111%2Fbjh.12659&file=bjh12659-sup-0001-TableS1-S8.xlsx
National Cancer Registry in Japan	2014-2015	Japan. Available from https://ganjoho.jp/reg_stat/statistics/data/dl/excel/cancer_incidence47pref(2014-2015).xls
National Cancer Registry in Japan	2016-2019	Japan. Available from: https://ganjoho.jp/reg_stat/statistics/data/dl/excel/cancer_incidenceNCR(2016-2019).xls (released Aug 2022)
KCCR	2001-2012	South Korea. Available from: https://www.ncbi.nlm.nih.gov/pmc/articles/PMC5784621/table/t1-crt-2017-093/ (published online Mar 2017)
NHID	2011-2015	South Korea. Available from: https://www.ncbi.nlm.nih.gov/pmc/articles/PMC7577801/figure/f1-crt-2020-089/ (published online May 2020)

KCCR, Korean Central Cancer Registry; NHID, National Health Information Database.

Table S2. Case numbers of FL among Taiwan, Japan and South Korea between 2001 and 2019.

	Taiwan			Japan			Korea[13]	Korea[2]
Year	Overall	Male	Female	Overall	Male	Female	Overall	Overall
2001	--	--	--	232	110	122	74	--
2002	118	68	50	287	149	138	89	--
2003	158	90	68	352	177	175	92	--
2004	144	80	64	425	218	207	77	--
2005	174	104	70	483	266	217	69	--
2006	183	102	81	504	230	274	102	--
2007	193	100	93	671	331	340	107	--
2008	169	91	78	790	389	401	124	--
2009	205	115	90	--	--	--	128	--
2010	226	104	122	--	--	--	132	--
2011	238	123	115	--	--	--	175	199
2012	249	136	113	--	--	--	196	197
2013	239	130	109	--	--	--	--	267
2014	263	127	136	6875	3227	3648	--	293
2015	290	145	145	7202	3379	3823	--	288
2016	319	169	150	8357	3895	4462	--	--
2017	296	139	157	8830	4222	4608	--	--
2018	362	170	192	9089	4371	4717	--	--
2019	405	188	217	9378	4365	5013	--	--

Table S3. Japan-to-Taiwan incidence rate ratios by gender, 2012–2019

Year	IRR (95% CI) Overall	IRR (95% CI) Male	IRR (95% CI) Female
2002	1.14 (0.92, 1.41)	1.04 (0.78, 1.38)	1.22 (0.88, 1.69)
2003	0.86 (0.72, 1.04)	0.79 (0.61, 1.01)	0.96 (0.73, 1.27)
2004	1.10 (0.91, 1.32)	1.05 (0.81, 1.35)	1.16 (0.87, 1.53)
2005	1.08 (0.91, 1.28)	1.01 (0.81, 1.27)	1.20 (0.91, 1.57)
2006	1.07 (0.90, 1.27)	0.85 (0.67, 1.07)	1.34 (1.05, 1.72)
2007	1.33 (1.13, 1.56)	1.32 (1.06, 1.65)	1.37 (1.09, 1.73)
2008	1.83 (1.55, 2.16)	1.72 (1.37, 2.16)	1.94 (1.52, 2.47)
2014	3.32 (2.93, 3.75)	3.27 (2.74, 3.90)	3.36 (2.83, 3.98)
2015	4.19 (3.73, 4.71)	3.00 (2.54, 3.54)	3.30 (2.79, 3.89)
2016	4.38 (3.92, 4.90)	2.90 (2.49, 3.38)	3.71 (3.15, 4.37)
2017	5.06 (4.51, 5.69)	3.80 (3.21, 4.50)	3.76 (3.21, 4.41)
2018	3.22 (2.90, 3.57)	3.28 (2.82, 3.83)	3.18 (2.75, 3.68)
2019	2.95 (2.67, 3.26)	2.90 (2.50, 3.35)	3.02 (2.64, 3.46)

IRR, incidence rate ratio; CI, confidence interval
